# Single‐cell transcriptome analysis reveals defective decidua stromal niche attributes to recurrent spontaneous abortion

**DOI:** 10.1111/cpr.13125

**Published:** 2021-09-21

**Authors:** Lili Du, Wenbo Deng, Shanshan Zeng, Pei Xu, Lijun Huang, Yingyu Liang, Yang Wang, Hui Xu, Jingman Tang, Shilei Bi, Lizi Zhang, Yulian Li, Luwen Ren, Lin Lin, Weinan Deng, Mingxing Liu, Jingsi Chen, Haibin Wang, Dunjin Chen

**Affiliations:** ^1^ Department of Obstetrics and Gynecology Key Laboratory for Major Obstetric Diseases of Guangdong Province The Third Affiliated Hospital of Guangzhou Medical University Guangzhou China; ^2^ Key Laboratory of Reproduction and Genetics of Guangdong Higher Education Institutes Guangzhou China; ^3^ Guangdong Engineering and Technology Research Center of Maternal‐Fetal Medicine Guangzhou China; ^4^ Department of Obstetrics and Gynecology The First Affiliated Hospital of Xiamen University Xiamen University Xiamen China; ^5^ Key Laboratory of Reproductive Health Research Fujian Province University School of Medicine Xiamen University Xiamen China; ^6^ Department of Obstetrics and Gynecology Nanfang Hospital Southern Medical University Guangzhou China

## Abstract

**Objectives:**

Successful pregnancy involves the homeostasis between maternal decidua and fetoplacental units, whose disruption contributes to compromised pregnancy outcomes, including recurrent spontaneous abortion (RSA). The role of cell heterogeneity of maternal decidua in RSA is yet to be illustrated.

**Materials and methods:**

A total of 66,078 single cells from decidua samples isolated from patients with RSA and healthy controls were analysed by unbiased single‐cell RNA sequencing (scRNA‐seq).

**Results:**

Our scRNA‐seq results revealed that stromal cells are the most abundant cell type in decidua during early pregnancy. RSA samples are accompanied by aberrant decidualization and obviously obstructed communication between stromal cells and other cell types, such as abnormal activation of macrophages and NK cells. In addition, the over‐activated TNF superfamily member 12 (TNFSF12, TWEAK) and FASLG in RSA are closely related to stromal cell demise and pregnancy failure.

**Conclusions:**

Our research reveals that the cell composition and communications in normal and RSA decidua at early pregnancy and provides insightful information for the pathology of RSA and will pave the way for pregnancy loss prevention.

## Graphical abstract

Recurrent spontaneous abortion (RSA), characterized by pregnancy loss before 20 weeks of gestation more than twice, is an intricated pregnancy complication with enigmatic underlying mechanism ascribes to its complex pathogenesis. The homeostasis between the developing foetus and maternal decidua is critical for pregnancy maintenance. By exploring the cell heterogeneity in normal and RSA decidua utilizing scRNA‐Seq, we unravel the discrepancies in cell composition and communications in these two distinct deciduae. Our investigations uncover that stromal cells are the most abundant cell populations in the decidua, with three different subpopulations at various decidualization stages and two fibroblasts. There are two separated trajectories of stromal cell decidualization marked by *PLA2G2A* and *WNT4*. As the most abundant cell population in the decidua, the stromal cells dominate the communications with other cell types, including endothelial cells, macrophages, uNK cells and perivascular cells. Compared with normal decidua, decidualized stromal cells are overtly decreased in RSA decidua with augmented macrophages. In addition, we present some previously unappreciated signaling pathways among different cells types in decidua and also depict the remarkably changed communications between normal and RSA decidual. The aberrant activated TWEAK and FASLG in RSA are considered to be potential reasons for stromal cells demise and pregnancy failure. Our research reveals the cell composition and communications in normal and RSA decidua at early pregnancy and provides insightful information for the pathology of RSA and will pave the way for pregnancy loss prevention.

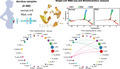

## 1 INTRODUCTION

Recurrent spontaneous abortion (RSA), experienced by 5% of fertile couples, is a pregnancy complication with an elusive underlying mechanism due to multiple reasons, including genetic predisposition, inflammation, immunology imbalance, maternal ageing, hormonal imbalance and environmental stresses.^1^, ^2^, ^3^ The logistical and ethical difficulties in accessing meaningful samples are the major limitations for RSA studies attributing to intangible pathogenesis. The availability of sufficient samples from diverse groups with varied backgrounds will greatly advance the mechanistic study of RSA.

Successful pregnancy involves the participation of maternal decidua and fetoplacental units with many other cell types, as revealed by single‐cell sequencing.^4^, ^5^ While the role of decidua in pregnancy maintenance is largely neglected, there is an increasing interest in the role of decidua in orchestrating the homeostatic balance between the mother and foetus, and studies suggest that the decidua is a critical regulator in the cross‐talk between foetus and decidua by secreting cytokines, morphogens and signalling molecular to safeguard foetal development.^6^, ^7^, ^8^, ^9^ Defective decidualization can lead to aberrations in placentation and adverse pregnancy outcomes. Failed decidualization is a vital contributor to downregulated cytotrophoblast invasion in severe pre‐eclampsia.^10^, ^11^, ^12^, ^13^ However, the clear communications between different cell types are not yet clear.

The maternal immune status also plays a critical role in the process of pregnancy. The distinct immune milieu from the first trimester to the third trimester suggests that the local immune niche at the maternal‐foetus interface is finely orchestrated to guarantee the growth of the developing foetus. There are multiple immune cells in the maternal‐foetal interface encompassing Natural killer (NK) cells, Innate lymphoid cells (ILCs), macrophages, T cells and others.^14^ NKs are essential for vessel remodelling and embryo nutrition mediated by pleiotrophin (PTN) and osteoglycin (OGN).^15^ But the function of other immune cells is not clear for foetal growth, and their relationship with RSA is also ambiguous. While the cell heterogeneity of the uterus was recognized in both humans and mice,^4^, ^16^, ^17^ the cell composition and communication between normal and RSA during pregnancy remain enigmatic.

In this study, scRNA‐seq is leveraged to unravel the cell heterogeneity of maternal decidua in normal and RSA groups in the first trimester. We observe that stromal cells are the most abundant cell type and that disrupted decidualization is also present in RSA samples. The communication between stromal cells and other cell types is obstructed in RSA samples. Besides, the aberrant activation of macrophages and NK cells is also observed in RSA samples. Collectively, our study elucidates the role of decidualized stromal cells, macrophages and NK cells in normal pregnancy as well as its role in RSA. The resulting findings reveal a potential mechanism of RSA pathogenesis and provide insightful information for the development of RSA and pave the way for RSA prevention.

## 2 METHODS

### 2.1 Sample collection

All tissue samples used for this study were obtained with written informed consent from all participants. The study was approved by the Medical Ethics Committee of The Third Affiliated Hospital of Guangzhou medical university, Medical Research (No. 20170126). Patients with two or more previous spontaneous unexplained abortions, normal karyotype of parents and abortus, and absence of uterine malformation, endocrine, metabolic, autoimmune diseases or infection were enrolled in the RSA group (6 for single‐cell RNA sequencing, 19 for quantitative reverse‐transcriptase polymerase chain reaction (qRT‐PCR)). Women who underwent elective termination of normal pregnancies without a history of miscarriages were eligible as healthy controls (5 for single‐cell RNA sequencing, 20 for qRT‐PCR). The demographic characteristics of participants in the RSA group and the normal group used for single‐cell RNA sequencing are displayed in Table S1. Samples of the RSA group were collected from women with RSA by ultrasound‐guided curettage immediately after the diagnosis of missed abortion. Decidua was identified macroscopically, washed in phosphate‐buffered saline to remove excess blood, and used for the single‐cell isolation as described below.

### 2.2 Isolation of decidual cells

Primary decidual cells were isolated as previously described from freshly collected decidual tissue.^18^ Briefly, decidua was rinsed several times with PBS until removal of obvious blood clots; then was cut into small pieces, which were digested with collagenase Type IV (0.5 mg/ml, C5138; Sigma‐Aldrich) and DNase I (0.1 mg/ml, DN25; Sigma‐Aldrich) for 30 min. The released decidual cells were filtered through 70 and 40 µm mesh sieves, centrifuged, and resuspended in 5 ml of red blood cell lysis buffer (Invitrogen, 00‐4300) for 10 min to exclude any remaining red blood cells. Finally, the pelleted decidual cells were resuspended in PBS and then were used for single‐cell 3′‐cDNA library preparation followed by 10× Genomics Chromium Single‐Cell 3′ reagent Kits protocol.

### 2.3 Single‐cell RNA‐seq data processing

Single‐cell libraries were sequenced on Illumina HiSeq X Ten instruments using 150 nt paired‐end sequencing. Reads were processed using the Cell Ranger 4.0.0 pipeline with the default and recommended parameters. FASTQs generated from the Illumina sequencing output were aligned to the human reference genome (GRCh38) using the STAR algorithm. Next, Gene‐Barcode matrices were generated for each sample by counting unique molecular identifiers (UMIs) and filtering non‐cell associated barcodes. Finally, the gene‐barcode matrix containing the barcoded cells and gene expression counts were generated.

This output was then imported into the Seurat (v3.0) R toolkit for quality control and downstream analysis of our single‐cell RNA‐seq data. All functions were run with default parameters unless specified otherwise. Low‐quality cells (<500 genes/cell) were excluded for each sample of all six RSA patients and five healthy controls. After filtering, a total of 66,078 cells were left for the following analysis. Finally, a filtered gene‐barcode matrix of all samples was integrated with Seurat v.3 to remove batch effects across different samples using the SCT method.

### 2.4 Identification of cell types and subtypes by Uniform Manifold Approximation and Projection (UMAP)

The Seurat package implemented in R was applied to identify major cell types. Highly variable genes were generated and used to perform PCA. Significant principal components were determined using JackStraw analysis and visualization of heatmaps focussing on PCs 1 to 30. PCs 1 to 30 were used for graph‐based clustering (at res = 0.6) to identify distinct groups of cells. We characterized the identities of cell types of these groups based on annotation results of singleR.

### 2.5 Cluster marker identification

The cluster‐specific marker genes were identified by running the FindAllMarkers function in the Seurat package to the normalized gene expression data. To identify differentially expressed genes between two clusters, we used the ‘findmarkers’ function. We used the R package clusterProfiler to perform biological process enrichment analysis with the top 20 differentially expressed genes in each cluster or subset.

### 2.6 Constructing cell trajectories

Trajectory analysis was performed separately for the cluster DS1, DS2 and DS3 cells using Monocle 2 (version 2.6.4). We then conducted differential gene expression analysis of the studied cells using the differentialGeneTest function to identify significant genes (BH‐corrected *p* < 0.05), and cell ordering was performed on these genes in an unsupervised fashion. Trajectory construction was then performed after dimensionality reduction and cell ordering with default parameters.

### 2.7 Cell‐cell communication analysis

To investigate potential interactions across different cell types in DS cells and immunity cells, cell‐cell communication analysis was performed using CellPhoneDB, a publicly available repository of curated receptors and ligands and their interactions. CellPhoneDB analysis was performed using the CellPhoneDB Python package (1.1.0). Enriched receptor‐ligand interactions between two cell types were derived based on the expression of a receptor by one cell type and the expression of the corresponding ligand by another cell type. Then, we identified the most relevant cell‐type‐specific interactions between ligands and receptors, and only receptors and ligands expressed in the cells in the corresponding subclusters were considered.

### 2.8 CellChat

To further analyse and compare the intercellular communication differences between RSA and normal decidua samples, CellChat, an open‐source R package (https://github.com/sqjin/CellChat) was used for RSA and normal decidua scRNA‐seq data. First, we inferred intercellular communications among DSC and other cell subsets for the RSA and normal datasets separately and then analysed them together via joint manifold learning and classification of the inferred communication networks based on their functional similarity.

### 2.9 SCENIC

Transcripts factor analysis was performed using SCENIC, following an automated SCENIC pipeline using the expression matrices (http://scenic.aertslab.org). The key regulators of DS, Macro, NK and Endo subsets in normal samples were identified.

### 2.10 RNA extraction and quantitative reverse‐transcriptase polymerase chain reaction analyses

Total RNA was extracted from decidua tissues obtained from RSA (*n* = 19) and normal (*n* = 20) groups using TRIzol (Takara, Japan), according to the manufacturer's protocol. Extracted RNA was diluted with DEPC‐treated water and quantified using a ratio of measurements at 260 and 280 nm (Nanodrop; Thermofisher), then were reversed to cDNA using a PrimeScript™ RT reagent Kit with gDNA Eraser (Takara, China) according to the manufacturer's instructions. The real‐time PCR system used RR420A TB Green™ Premix Ex Taq™ (Tli RNaseH Plus) (Takara, Japan), with primer sequences provided in Table S4. All real‐time PCR (qPCR) reactions were carried out using the Q3 Real‐Time System (Applied BioSystems). The results were normalized to the expression of SDHA. Relative fold change in gene expression were calculated using the 2^−ΔΔCt^ method, normalized with respective controls.

### 2.11 IHC analysis and Western blot analysis

For immunohistochemistry (IHC), tissue samples were fixed with 10% buffered formalin at room temperature for 6 h. Samples were embedded in paraffin. After routine rehydration and antigen retrieval, 5‐μm paraffin sections were stained for HE or with the primary antibodies (Table S5). Macrophages and dNKs were identified by anti‐CD68 and anti‐CD56 immunostaining, respectively. The sections were further incubated with HRP‐conjugated secondary antibodies and visualized with a DAB solution containing 0.03% H_2_O_2_. Negative/normal controls were performed by replacing the specific antibody with rabbit or mouse IgG. Whole lysates from tissues were extracted with RIPA buffer containing protease inhibitor cocktail (Sigma‐Aldrich). Protein concentrations were determined using the BCA™ Protein Assay Kit (Pierce). Western blotting was performed as described previously. Antibodies used for western blotting include Fn14 and FAS (Table S6).

### 2.12 In‐situ hybridization and Immunofluorescence staining

Frozen sections from normal decidua were used for In‐situ hybridization and immunofluorescence (IF) staining. Digoxigenin (DIG)‐labelled IGFBP1 probes were generated according to the manufacturer's protocol (Roche). In‐situ hybridization with DIG‐labelled probes was performed as described.^19^ Antibodies used for IF staining include PR and HAND2 (Table S5).

## 3 RESULTS

### 3.1 Single‐cell atlas in normal and RSA decidua

**FIGURE 1:**
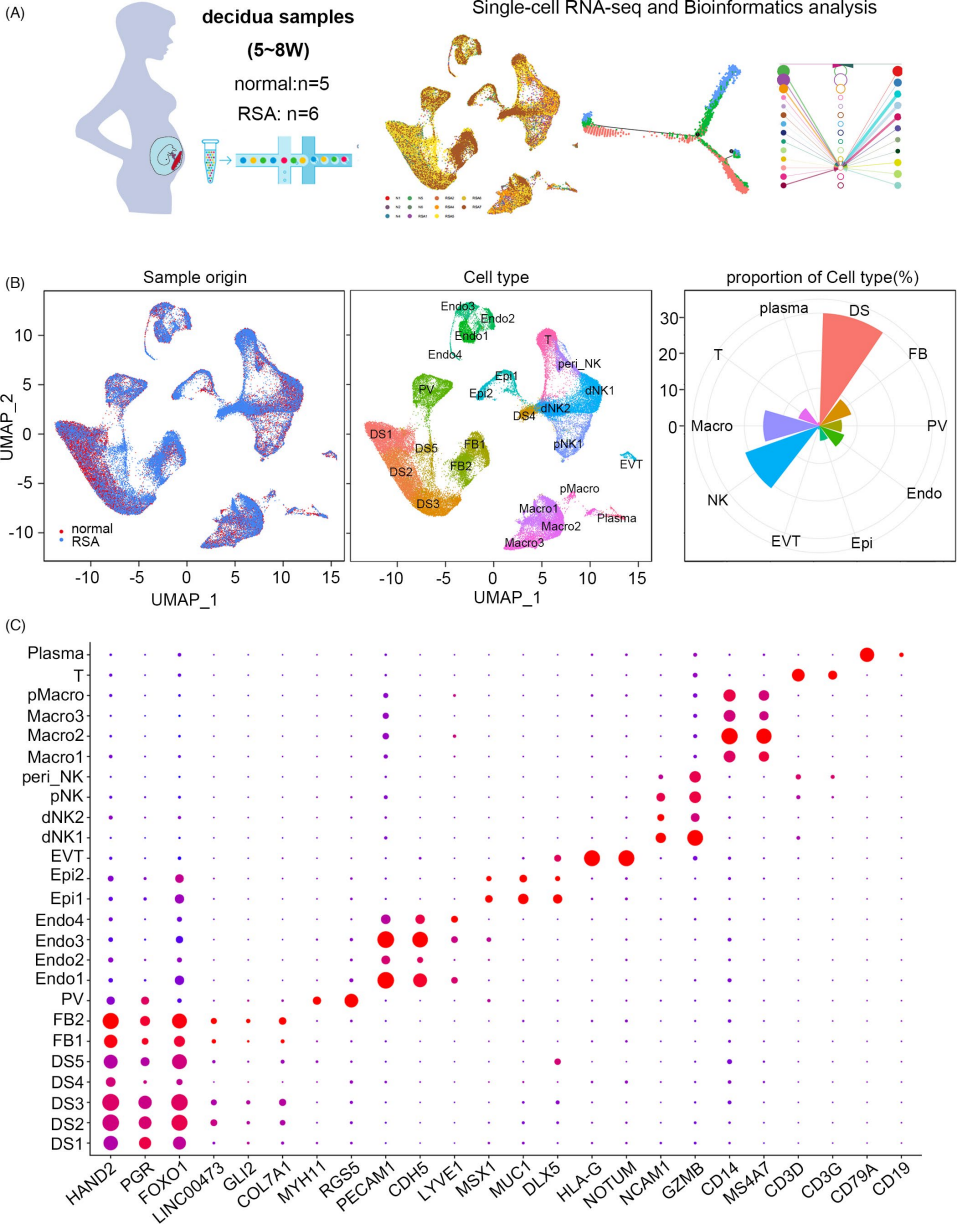
Overview of the 66,078 single cells from normal or RSA decidua. (A) Summary of the sample origins and analysis workflow. The decidua tissue was collected and processed into a single‐cell suspension and single‐cell RNA sequencing was performed using the 10× Genomics platform followed by bioinformatics analysis. (B) UMAP of the 66,078 cells with its sample type of origin (RSA or normal), the associated cell type and the proportion of each cell type in decidua samples. (C) Dotplot map showing the expression of classical cell type‐specific marker genes in each cluster

Cumulative evidence suggests that the decidua plays a critical role in pregnancy haemostasis by regulating programmed decidual senescence and local inflammation.^20^ Furthermore, the endothelial cells in the decidua act as sentinels and are the first line of defence to combat infection.^21^ Numerous immune cells are indispensable for immune balance in the maternal‐foetus interface during pregnancy.^22^ However, the detailed mechanism of each cell type in the decidua during pregnancy is largely unknown. To decipher the heterogeneity of the decidua, single‐cell RNA‐seq was performed in first‐trimester (5–8 weeks of gestation, 6.20 ± 1.09 weeks) decidua from five normal samples using 10× Genomics, as well as six RSA (5–8 weeks of gestation, 6.83 ± 0.75 weeks) samples who experienced more than two unexplained miscarriages without foetal chromosomal abnormality as confirmed by karyotype or array comparative genomic hybridization (CGH) analysis (Figure 1A and Figure S1A, Table S1).

After quality filtering and batch correction, graph‐based clustering was performed to group a total of 66,078 cells composed of 31,140 cells from normal decidua and 34,938 cells from RSA decidua, respectively, according to their gene expression profile (Table S2). There were 10 major clusters (containing 25 subclusters) in both RSA and control samples, including decidualized stromal cells (DS, including DS1–DS5), fibroblasts (FB1 and FB2), natural killer cells (decidual NK1 (dNK1), dNK2, proliferating NK (pNK) and peripheral NK (peri_NK)), macrophages (Macro1, Macro2, Macro3 and proliferating Macro (pMacro)), T cells, plasma cells, perivascular cells (PV), endothelial cells (including Endo1, Endo2, Endo3 and Endo4), trophoblasts and epithelial cells (including Epi1 and Epi2). Clusters were visualized using UMAP (Uniform Manifold Approximation and Projection) (Figure 1B) and were annotated based on marker genes (Figure 1C, Table S3). Among them, decidual stromal cells were the most abundant cells, followed by NK cells, macrophages, T cells and others (Figure 1B). Similar cell composition was observed in normal and RSA decidua (Figure S1B).

### 3.2 The characterization of different stromal cells in decidua

**FIGURE 2:**
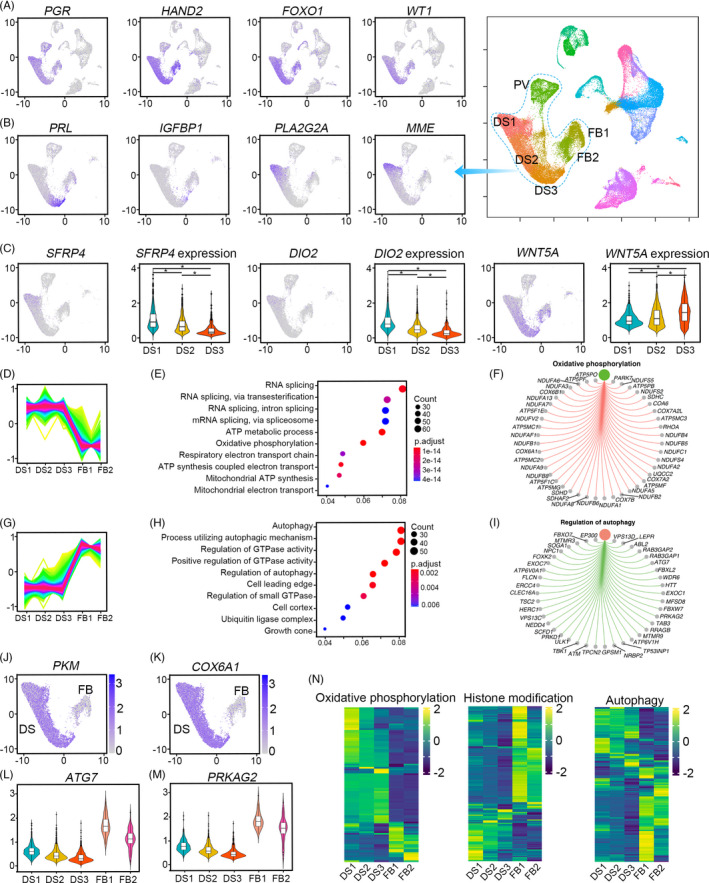
Characterization of different stromal cells in normal decidua samples. (A) Feature plot showing the expression of *PR*, *HAND2*, *WT1* and *FOXO1* in decidua stroma cells; (B) Feature plot showing the expression of *PRL*, *IGFBP1*, PLA2G2A and MME among different DS subsets; (C) Feature plot and violin plot showing the expression of *SFRP4*, *DIO2* and *WNT5A* among distinct stromal subsets; (D–F) The genes with higher levels in DS1, DS2 and DS3 than FB1 and FB2, based on the results of TCseq (D); the functional enrichment of these genes (E); and oxidative phosphorylation associated genes in these DSs specific genes (F); (G–I) The genes with higher levels in FBs than DSs, based on the results of TCseq (G); The functional enrichment of these FBs specific genes (H) and autophagy‐associated genes in FBs specific genes (I). (J, K) Feature plot showing the higher expression of *PKM* and *COX6A1* in DS subsets with lower expression in FB subsets. (L, M) Violin plot showing the higher expression of *ATG7* and *PRKAG2* in FB subsets with lower expression in DS subsets. (N) Heat map showing DS subsets were enriched for oxidative phosphorylation regulating genes and FB subsets were enriched for autophagy and histone modification associated genes

After embryo implantation, the stromal cells undergo extensive decidualization to support the growth of the developing foetus. These stromal cells were identified by the expression of progesterone receptor (*PGR*), heart and neural crest derivatives expressed 2 (*HAND2*), WT1 transcription factor (*WT1*) and forkhead box O1 (*FOXO1*) (Figure 2A and Figure S2A). Normal decidua mainly contains three types of stromal cells (DS1, DS2 and DS3) and two FBs (FB1 and FB2) with a small DS4 and DS5. The different types of stromal cells and their potential function attracted our attention. We first explored the expression profile of stromal cells DS1, DS2 and DS3 due to their abundance in the normal decidua. DS1, DS2 and DS3 were characterized by gradually increased expression of insulin‐like growth factor‐binding protein 1 (*IGFBP1*) and prolactin (*PRL*), with the highest expression in DS3 (Figure 2B and Figure S2B). There were some genes specifically expressed in DS1, including phospholipase A2 group IIA (*PLA2G2A*), membrane metalloendopeptidase (*MME*), secreted frizzled‐related protein 4 (*SFRP4*), secreted frizzled‐related protein 1 (*SFRP1*) and iodothyronine deiodinase 2 (*DIO2*), while Wnt family member 5A (*WNT5A*) was highly expressed in DS2 and DS3 (Figure 2B,C, Figure S3A,B). MME, known as CD10, is a well‐recognized human endometrium stromal cell marker.^23^, ^24^ The expression of CD10 was only observed in non‐pregnant endometrium but dramatically decreased in the decidua of early gestation,^25^ which is consistent with our results that CD10 is mainly reduced in DS3. PLA2G2A, a member of the phospholipase A2 family (PLA2) producing biologically active lipid mediators such as lysophosphatidic acid (LPA) and arachidonic acid (AA), is a critical enzyme for inflammatory processes and the regulation of the phospholipid metabolism.^26^, ^27^ Intracellular PLA2 was reported to restrict Wnt signalling during haemostasis in the small intestine.^28^ This speculation was also supported by the expression of SFRP1 and SFRP4, critical inhibitors of Wnt signalling directed differentiation.^29^, ^30^ These reports suggested that DS1 stromal cells were less decidualized, but DS3 underwent extensive decidualization.

In decidualization, 3,5,3′‐triiodothyronine (T3) is inactivated in decidualized stromal cells to facilitate decidualization. DIO2, a critical enzyme for producing active T3 from 3,5,3′,5′‐tetraiodothyronine (T4), is highly expressed in sub‐epithelial stromal cells before embryo implantation in mice; while its expression is dramatically decreased after the initiation of decidualization.^31^ The downregulation of *DIO2* in in‐vitro decidualized endometrial stromal cells as examined by real‐time PCR further corroborated this discovery (Figure 2C and Figure S3C). Decreasing DIO2 observed in both mice and humans suggests DIO2 positive cells were proved to be expressed in stromal cells with less decidualization. This speculation was also supported by the expression of WNT signalling with highly expressed *IGFBP1*, *PRL* and *WNT5A* in DS3 (Figure 2B,C). In addition, DS3 also expressed interleukin 1 beta (*IL1B*), microfibril associated protein 4 (*MFAP4*), a regulator of cell cycle (*RGCC*) and C‐X‐C motif chemokine ligand 14 (*CXCL14*) (Figure S3D). We also noticed that DS2 had a higher expression of *LINC00473* (Figure S3E), a long non‐coding RNA regulated by cAMP during endometrial decidualization.^32^ Accordingly, there were at least three different stromal cells dependent on their differentiation degree.

To globally depict the signature of these stromal cells thoroughly, a profile of these three DSs and two FBs were clustered based on their gene expression pattern using TCseq analysis (Figure S3F). The genes highly expressed in all DSs showed significant enrichment of genes associated with oxidative phosphorylation, glycolysis and mitochondria(Figure 2D–F), such as pyruvate kinase M1/2 (*PKM*) and cytochrome c oxidase subunit 6A1 (*COX6A1*) (Figure 2J,K). Genes highly expressed in FBs were predisposed to slow down cell metabolism with elevated levels of some autophagy‐associated genes (Figure 2G–I), such as autophagy‐related 7 (*ATG7*) and protein kinase AMP‐activated non‐catalytic subunit gamma 2 (*PRKAG2*, AMPK gamma subunit) (Figure 2L,M). To further confirm these results, all genes related to autophagy and oxidative phosphorylation were visualized in DSs and FBs (Figure 2N). Most autophagy‐associated genes were specifically expressed in FBs; whereas most oxidative phosphorylation genes were expressed in DSs. We also noticed that some histone modification enzymes were significantly enriched in FBs (Figure 2N). Moreover, a few DS4 (246) cells remained in the normal decidua. Compared to DS1, DS2 and DS3 cells, DS4 cells expressed higher epithelial markers, such as *PAEP* and *KRT18*, and lower mesenchymal markers, such as *ZEB1* and *TWIST2* (Figure S3G).

### 3.3 Defective stromal decidualization contributes to RSA

**FIGURE 3:**
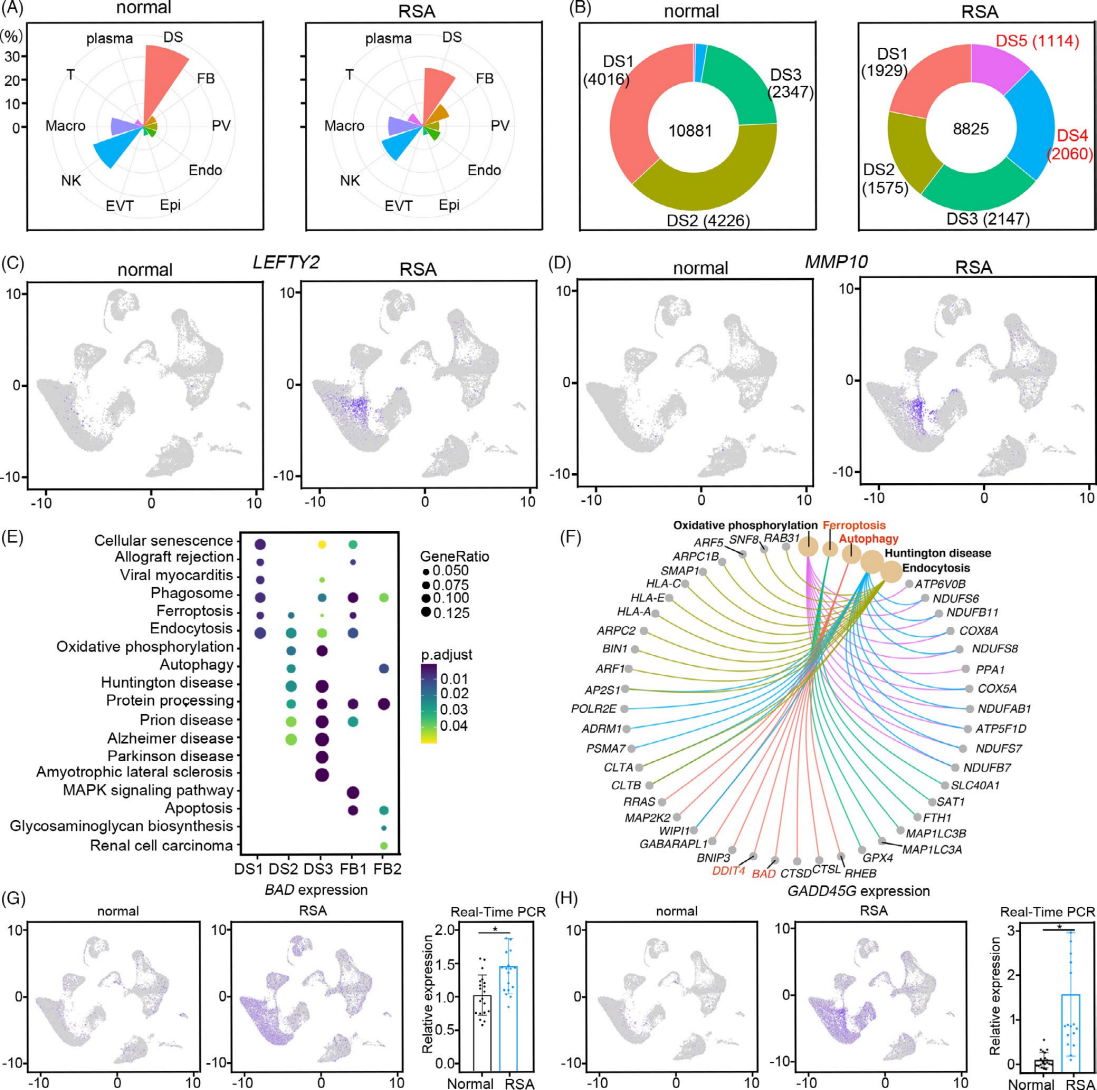
Defective stromal decidualization contributes to RSA. (A) The proportion of each cell type in normal and RSA decidua; (B) The cell number of each DS subset in different groups; (C, D) Feature plot showing the expression of *LEFTY2* and *MMP10* in DS5 subsets in normal and RSA decidua; (E, F) Dot plot showing the function enrichment analysis of DEGs among each DS subset between normal and RSA decidua and oxidative phosphorylation, ferroptosis, autophagy, huntington disease and endocytosis associated genes; (G, H) Feature plot and boxplot plot showing the expression of *BAD* and *GADD45G* in normal and RSA decidua by scRNA‐seq and the confirmation by real‐time PCR

As the normal decidual stromal cells were delineated, we explored the difference between normal and RSA decidua. Notably, the number of DS cells was significantly decreased in RSA decidua (Figure 3A,B). It was very interesting that the populations of DS1 and DS2 in RSA were significantly decreased when compared with normal stromal cells. Meanwhile, there was a new cell population emerging in the RSA group, DS5 (Figure 3B, Figure S4A), as outlined by the expression of matrix metallopeptidase 10 (*MMP10*) and left‐right determination factor 2 (*LEFTY2*) (Figure 3C,D). *LEFTY2* is a member of the transforming growth factor (TGF)‐β family and an endometrium bleeding associated factor, and it is upregulated in the late luteal phase of the menstrual cycle, coinciding with the closure of the window of implantation.^33^ Genes highly expressed in DS5 were enriched in cell apoptosis and senescence pathways, such as growth arrest and DNA damage‐inducible gamma (*GADD45G*) and insulin‐like growth factor‐binding protein 3 (*IGFBP3*) (Figure S4B,C). These data suggested that the stromal cells underwent abnormal development in RSA decidua.

Function enrichment analysis suggested that the upregulated genes in RSA decidual stromal cells were mainly associated with cellular senescence, ferroptosis, apoptosis, endocytosis and autophagy (Figure 3E,F). Among them, BCL2 associated agonist of cell death (*BAD*), an apoptosis‐associated gene, was widely expressed in all RSA DSs with very low expression in normal decidual, as well as *GADD45G* (Figure 3G,H). Our real‐time PCR results also confirmed the expression pattern of both genes in RSA and control groups (Figure 3G,H).

**FIGURE 4:**
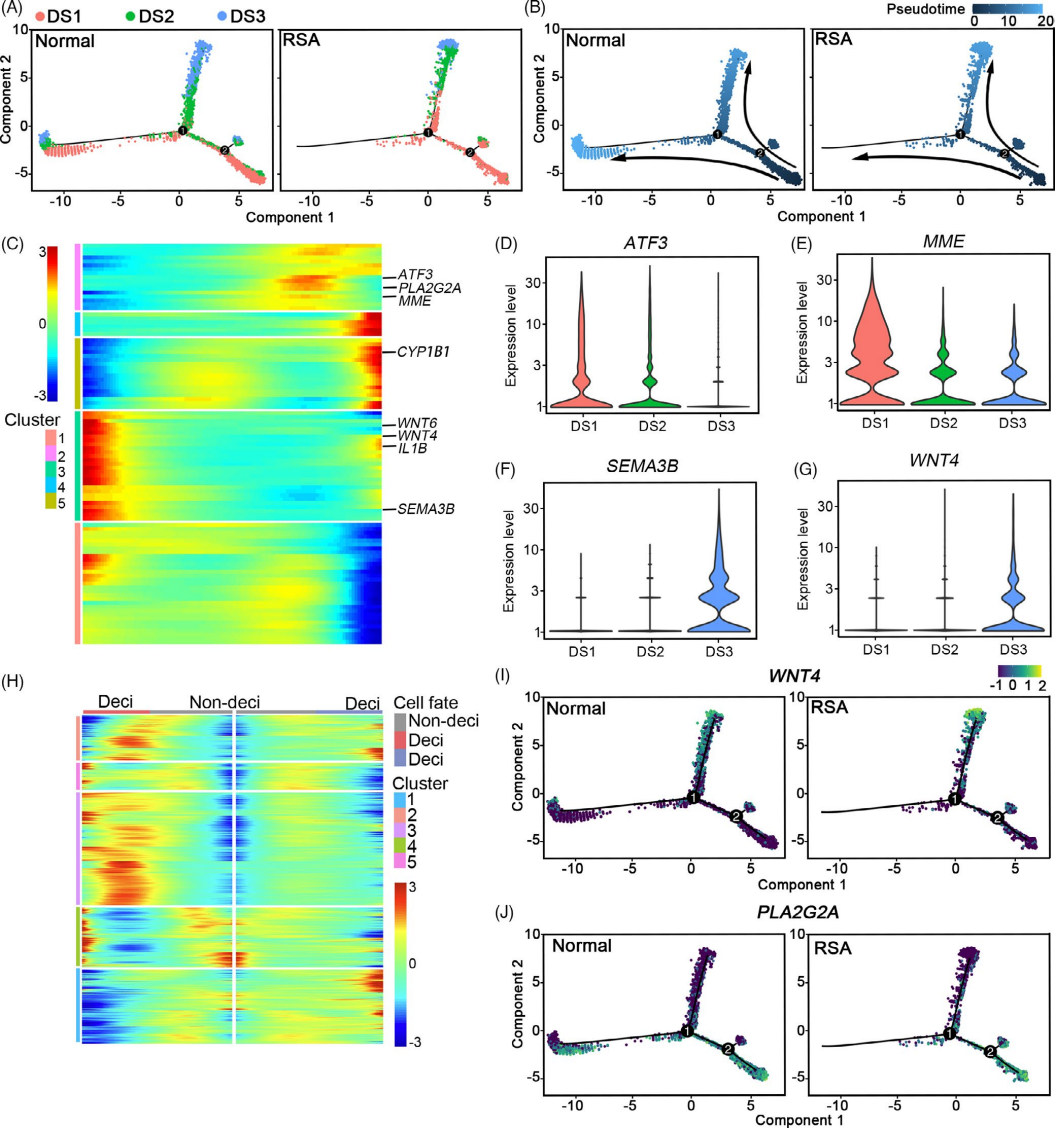
Stromal cell development progress based on molecular trajectory analysis. (A, B) Pseudotime ordering of DS1, DS2 and DS3 in normal and RSA samples by monocle analysis. Each dot represents one cell and each branch represents one cell state. A was labelled with cell states and B was labelled with developmental pseudotime. (C) Heatmap showing the significant enriched gene dynamics along pseudotime; (D–G) Violin plot showing the expression profile of *ATF3*, *MME*, *SEMA3B* and *WNT4* among normal DS subset; (H) Heatmap showing the decidualization related gene dynamics among different cell fates by branch analysis; (I, J) The expression of *WNT4* and *PLA2G2A* along pseudotime among DS subsets in the normal and RSA group

The increased expression of genes related to cell death in RSA decidua indicated that the stroma underwent unusual development. To confirm this, we investigated the stromal cell development progress by molecular trajectory analysis. In normal decidua, the undifferentiated stromal cells (DS1) developed to extensively differentiated decidualized cells (DS3) as marked by *IGFBP1* and *PRL* through DS2 by two separated progress (Figure 4A,B). In RSA patients, the trajectory of decidualization was largely compromised, and one trajectory branch had almost disappeared (Figure 4A,B). In the pseudotime heatmap, activating transcription factor 3 (*ATF3*) and *MME* were highly enriched in the middle; *WNT6*, *WNT4*, semaphorin 3B (*SEMA3B*) and *IL1B* were enriched in one end and cytochrome P450 family 1 subfamily B member 1 (*CYP1B1*) was enriched in the other end (Figure 4C). The expression profile of *ATF3* and *MME* showed that they were highly expressed in undifferentiated stromal cells (Figure 4D,E), *SEMA3B* and *WNT4* were expressed in the decidualized DS3 (Figure 4F,G). The branch analysis also showed that *WNT4* and *PLA2G2A* were critical branch‐relevant genes (Figure 4H). Some cells were decidualized along with high expression of *WNT4*, while some cells highly expressed *PLA2G2A* (Figure 4I,J).

### 3.4 Cell connection in the decidual niche

**FIGURE 5:**
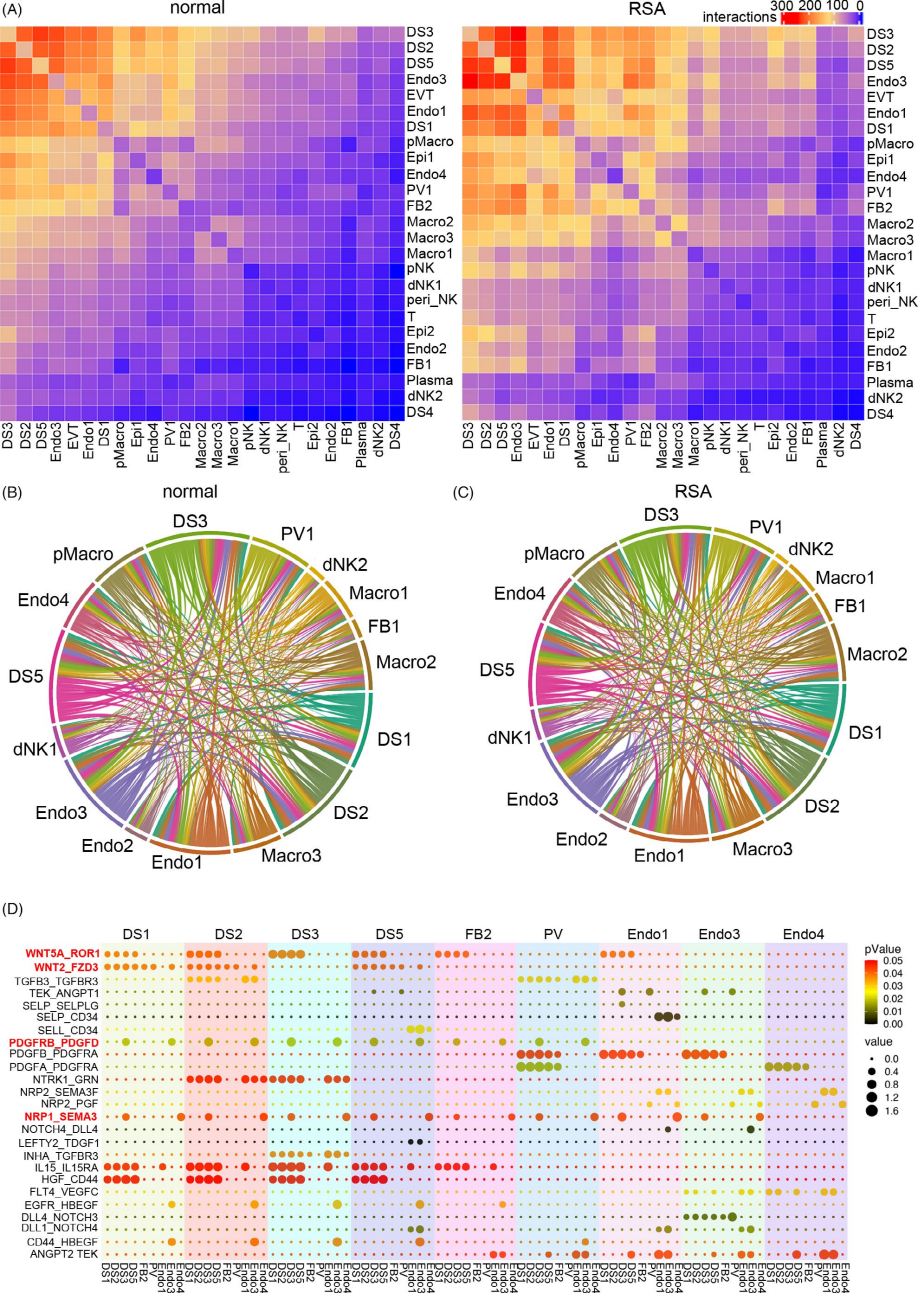
Cell connection among cell types in the decidua niche. (A) Heatmap showing the total number of interactions between cell types in normal and RSA samples utilizing CellPhoneDB; (B, C) The sending signalling and receiving signalling among different cell types in normal and RSA samples; (D) The detailed ligand‐receptor connections among DS, FB, PV and Endo subsets in normal samples. The top labelled subsets were as senders and the bottom subsets were as receivers. The colour darkness of dots represents the *p*‐values, and the size of the dots corresponds to the value of Ligand‐receptor interactions, scale on the right

Cell‐cell cross‐talk plays a critical role in haemostasis maintenance. It remains largely unknown how the defective decidualization in RSA disturbs local cell communications and ultimately leads to pregnancy loss. To delineate the interactions between different cell types, the interactions between stromal cells, macrophages, NKs, and other cells were investigated utilizing CellphoneDB. Our results showed that, in the normal decidua, DS3 stromal cells actively interacted with other stromal cells, especially DS2, endothelial cells (Endo 1 and Endo 3), inferred as EPH receptor B4 (*EPHB4*) positive endothelial cells in decidual veins (Figure S5A,B), EVT, PV and pMac (Figure 5A). There were almost 300 interactions between DS3 and DS2 and Endo3, but DS3’s connections with T cell, Epi2, Endo2, FB1, dNK2 and DS4 remained significantly less (Figure 5A). It was interesting that the overall cell‐cell interactions in RSA decidua were significantly increased (Figure 5A). For each cell type, the signallings sent or received were grossly similar between normal and RSA (Figure 5B,C). As the connection abundance between DSs and the endothelium was listed at the top, the detailed connections between these cell types were enumerated (Figure 5D). The WNTs communications were mainly presented between DSs and the endothelium. A novel mutual connection of SEMA3A (Semaphorin 3A)‐NRP1 (Neuropilin 1) was identified between the endothelium and DS cells (Figure 5D). The major function of SEMA3A was reported to inhibit angiogenesis of endothelial cells via neuropilins and plexins.^34^ As the expression of SEMA3A was observed in the proliferative phase of the endometrium,^35^ it was possible that the developing decidua resisted new blood vessel formation by SEMA3A secretion.

### 3.5 Cell communications in the decidual niche

**FIGURE 6:**
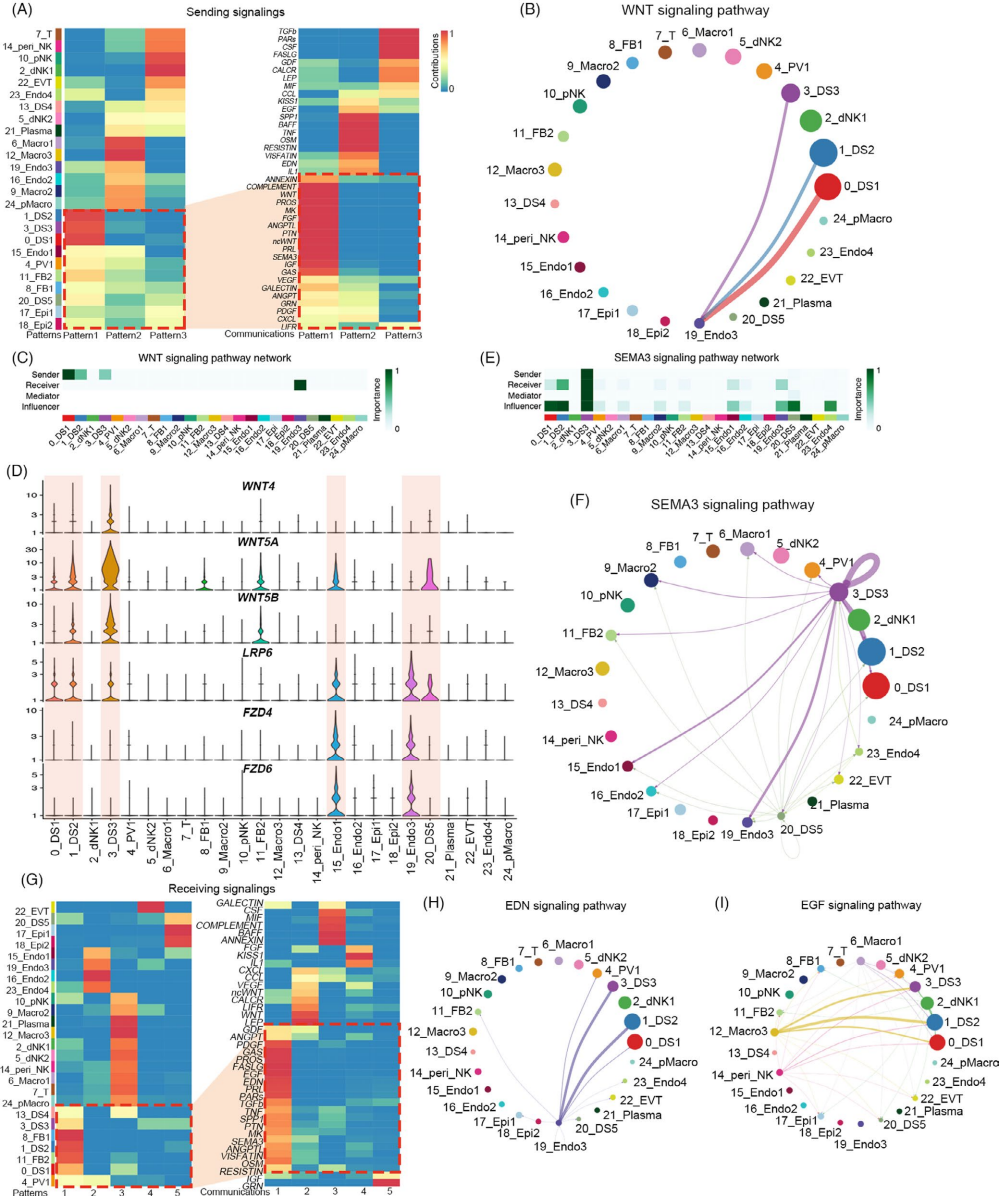
Cell‐cell cross‐talk between different cell types in normal decidua. (A) Heatmap showing the sending signal patterns of all cell types in normal decidua by CellChat analysis, and the first pattern included DS1, DS2, DS3, FB1, FB2, PV, Epi1, Epi2 and Endo1, with the secretion of WNT, ncWNT, SEMA3, PDGF, ANGPT (Angiopoietin), ANGPTL (Angiopoietin like) and PRL signalling; (B) Circle plot showing the inferred connection between canonical WNT signalling networks among DS subsets and endothelial cells in normal decidua; (C) Heatmap showing the relative importance of each cell type as sender and receiver for WNT signalling in normal decidua; (D) Violin plot showing the expression of major ligand‐receptors of WNT and ncWNT signalling pathways in normal decidua; (E) Heatmap showing the relative importance of each cell type as sender and receiver for SEMA3 signalling in normal decidua; (F) Circle plot showing the inferred SEMA3 signalling networks among different cell types in normal decidua; (G) Heatmap showing the receiving signal patterns of all cell types in normal decidua, and the first pattern included DS1, DS2, DS3, FB1, FB2 and PV, shared a very similar signalling pattern including EDN (Endothelin), EGF (Epidermal growth factor), PDGF and others; (H, I) Circle plot showing the inferred EDN and EGF signalling networks among different cell types in normal decidua

To globally distinguish the sending signalling and receiving signalling in different decidual cell types, CellChat was applied to investigate the cell‐cell cross‐talk.^36^ We first explored the sending signal of all cell types in the normal decidua. There were approximately three different types of sending signals, including DS1, DS2, DS3, FB1, FB2, PV, Epi1, Epi2 and Endo1 in the first pattern with the secretion of WNT, ncWNT, SEMA3, PDGF, ANGPT (Angiopoietin), ANGPTL (Angiopoietin like) and PRL (Figure 6A). Although DSs were featured by WNT secretion, targets of these WNTs were not clear. We showed that both canonical and non‐canonical WNTs were mainly produced by DSs and targeted different endothelial cells. The primary target of canonical WNTs was Endo3, while non‐canonical WNTs target Endo1, Endo2 and Endo3 (Figure 6B,C and Figure S6A). *WNT4*, *WNT5A* and *WNT5B*, which were the major players of ncWNT and WNT, were highly expressed in DSs. Their corresponding receptors LDL receptor‐related protein 6 (*LRP6*), frizzled class receptor 4 (*FZD4*) and *FZD6* were primarily expressed in different endothelium, with some LRP6 also expressed in DSs (Figure 6D). We also provided clear evidence that the SEMA3 generated by DSs, especially DS3, targeted endothelium and DSs (Figure 6E,F). This data suggested that comprehensive cross‐talks between DSs and the endothelium ensure normal foetal development.

For the receiving signalling, DS1, DS2, DS3, FB1, FB2 and PV shared a very similar signalling pattern, including EDN (Endothelin), EGF (Epidermal growth factor), PDGF and others, partially ascribing to a similar origin of these cell types (Figure 6G). We showed that Endo3 produced the most EDN targeting DS1, DS2, DS3 and FBs (Figure 6H and Figure S6B). This data further confirmed the closed interaction between stromal cells and endothelial cells. At the early stage of pregnancy, the stromal cells still underwent proliferation and growth, but the source of the growth signalling remains unclear. Here we revealed that multiple growth factors released by macrophages, including HB‐EGF, AREG (Amphiregulin) and EREG (Epiregulin) target *EGFRs* on DSs (Figure 6I and Figure S6C,D). Interestingly, PV cells expressed most stromal cell markers, such as *PGR*, *HADN2* and *WT1*. Our data also revealed that these perivascular cells intimately interacted with stromal cells by PDGF‐PDGFRs signalling pathways (Figure S6E,F). Overall, these data suggested decidual stromal cells functioned as a central hub with extensive communications with other cell types to guide foetal growth.

### 3.6 Derailed communications contributed to RSA

**FIGURE 7:**
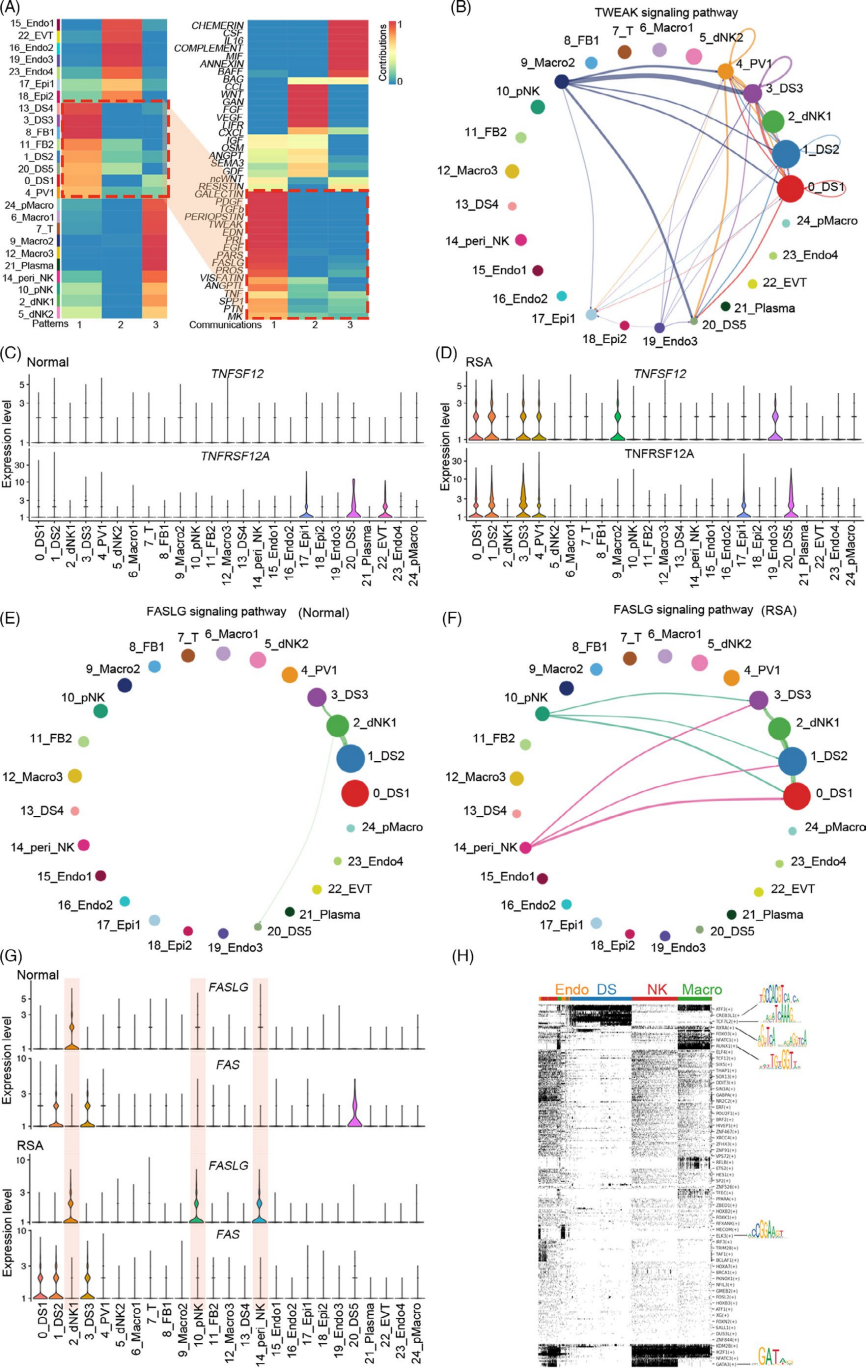
Derailed communications contributed to RSA. (A) Heatmap showing the receiving signal patterns of all cell types in RSA decidua by CellChat analysis; and the signalling received by DS1, DS2, DS3, DS4, DS5, FB1, FB2 and PV was clustered to the same one; (B) Circle plot showing the abnormal TWEAK signalling networks among different cell types in RSA decidua; (C, D) Violin plot showing the expression of ligand‐receptor pair of TWEAK (*TNFSF12* and *TNFRSF12A*) signalling among each cell type in normal and RSA decidua; (E, F) Circle plot showing FASLG signalling networks among different cell types in normal and RSA decidua; (G) Violin plot showing the expression of ligand‐receptor pair of FASLG (*FAS* and *FASLG*) signalling among each cell type in normal and RSA decidua; (H) Heatmap with binarized regulon activity showing the enriched regulatory landscape of DS, Macro, NK and Endo cell types in normal decidua by SCENIC. The column shows a single cell while the row shows regulons. For the regulons of particular interest, their representative binding motif was visualized in the right panel. ‘Black’ depicts active, while ‘white’ represents inactive

Given the tight interactions in different cell types, we were interested in the relationship between cell communications and clinical pregnancy loss. We first characterized the receiving signalling of RSA decidua. Similarly, the signalling received by DS1, DS2, DS3, DS4, DS5, FB1, FB2 and PV was clustered to the same cluster (Figure 7A). Except for PDGF, EDN, PRL and ANGPT, some signalling pathways were enriched in RSA decidua but not in the normal decidua. Among them, the TWEAK (TNF related weak inducer of apoptosis) signalling pathway was only enriched in RSA decidua with TWEAK (*TNFSF12*) from macrophages and DSs, and its receptor FN14 (*TNFRSF12A*) on different DSs (Figure 7B–D). Our real‐time PCR and western blotting results also confirmed that the levels of *TNFRSF12A* were higher in RSA decidua (Figure S7A–C). These data indicated that macrophages play an important role in RSA. We then inspected the difference of macrophages between normal and RSA decidua. There were four different macrophages in the decidua, with one proliferating group (Figure S8A,B). Among the three non‐proliferating macrophages, Mac1 was equipped with higher phagosome and proteoglycans activity, and Mac2 and Mac3 were featured with more robust antigen processing and presentation capability (Figure S8C,D). The population of Mac1 was increased in RSA compared with normal decidua with a similar number of other macrophages (Figure S8E). The molecular trajectory of macrophages in these two groups was also comparable (Figure S8F). Functional enrichment based on the differentially expressed genes uncovered the TNFα and NFkB signalling pathways were significantly upregulated in RSA Macro1 (Figure S8G,I). Moreover, phagosome and antigen processing and presentation pathways were enriched in RSA Macro3 upregulated DEGs (Figure S8H,I).

Another signalling pathway that was varied was the FASLG (Fas ligand) signalling pathway. There was a weak interaction between NK cells and DS2, DS3, DS5 decidual in the normal decidua, which indicated these stromal cells would be eliminated by NK‐derived cell death signal FASLG‐FAS signalling pathway (Figure 7E). DNK1 was the only cell type expressing *FASLG*, while *FAS* was exclusively expressed in DS2, DS3 and DS5 (Figure 7G). However, in RSA decidua, dNK1, pNK and peri_NK expressed high levels of *FASLG* with specific FAS expression in DS1, DS2 and DS3 (Figure 7F,G). Our real‐time PCR and WB results also confirmed the expression of FAS was more robust in RSA decidua (Figure S7D,E). This expression pattern suggested that activated NK cells may cause stromal cell demise.

To figure out the regulatory landscape of these four major cell types in the decidua, SCENIC was utilized to investigate the potential regulators of each cell type. CREB3L1 (cAMP responsive element binding protein 3 like 1) and TCF7L2 (Transcription factor 7 like 2) were significantly enriched in DSs (Figure 7H). The higher expression of *ATF3* and *WNTs* in DSs confirmed that the cAMP and WNT signalling pathways were critical regulatory factors for DSs. For macrophages, RXRA was significantly enriched. As RXRs were essential lineage determinants in peritoneal macrophages and osteoclasts,^37^ whether RXRs were also independent for decidual macrophage maintenance requires further investigation.

## 4 DISCUSSION

The role of decidua during early human pregnancy has been recognized recently. Still, the cellular and molecular mechanism and the detailed interaction between different cell types in the decidua remain elusive, ascribing to the ethical issue, the difficulty of accessibility of suitable tissue, and the lack of appropriate animal models. Our current study finds that there are different decidualized stromal cells in the human decidua, and each stromal cell poses its unique feature. The matured stroma cells are characterized by a profound secretory nature, including well‐established *IGFBP1* and *PRL*, which is encapsulated by many other studies.^38^, ^39^ Our investigation also found these matured decidualized stromal cells also communicate with other stromal cells, as well as endothelial cells and macrophages. PDGFA is a well‐known stromal cell‐specific secreting protein, which interacts with other cell types to maintain the stability of the microenvironment.^40^ This secretory nature of these decidualized cells comes with increased energy consumption and an adaptive metabolism change.

Our study also unravels that the FBs, undecidualized stromal cells in the endometrium, expressed DIO2 and some autophagy‐associated genes. These decidualization‐resistant cells in the endometrium are probably utilized to prevent uncontrolled invasion of EVT and are also responsible for rebuilding the endometrium after parturition. Our observations suggest the stromal cells should undergo sequential and sufficient decidualization to support foetal growth. The number of decidualized stromal cells, especially DS1 and DS2, in RSA decidua is remarkably diminished, indicating insufficient support of DSs in foetal development. Meanwhile, we also noticed that the number of DS5 is dramatically increased in RSA decidua. It is possible that at the early stage of decidualization development, some stromal cells do not differentiate into DS1 and DS2 appropriately but to DS5. In addition, a recent study reported highly proliferative mesenchymal cells (hPMC) were found in mid‐luteal human endometrium and hPMC may differentiate into dS3 cells in early pregnancy.^41^ It is unclear at present whether defective DS3 cells in RSA decidua in early pregnancy are associated with the loss of hPMC in the mid‐luteal endometrium, which is interesting and needs to be further studied. In summary, derailed stromal cell differentiation, contributing to abrupt decidual cell composition, creates unhealthy circumstances and ultimately leads to pregnancy loss.

The immune cells are dynamically changed during pregnancy. Decidual NK cells, which was the largest population of decidual leukocytes at the maternal‐foetal interface during early pregnancy, have abnormal properties, including subset composition, gene expression and function in patients with pregnancy loss.^42^, ^43^ Similar to Wang's study, we also found peri_NK (CD16+) and pNK (MKI67+) subsets in decidua tissues. Although the proportion of peri_NK and pNK was not increased in RSA samples according to our data, the more active signalling pathways of FASLG and TRAIL were exhibited in NK subsets of RSA groups. NK cell expression of FASL and TRAIL can contribute to apoptosis in their targets and NK killing.^44^, ^45^ uNK cells actively eliminate senescent stromal cells during decidualization.^46^ These findings support the defective stromal decidualization and compromised immune environment in the decidua from RSA patients. Immune profiling tests and personalization treatments at the time of implantation may help improve pregnancy outcome.^47^

DS dominates cell communications among cell types with the most abundant cell number and the highest activity as the primary cell type in the decidua. There are close interactions between stromal cells and endothelial cells mediated by endothelial EDN and its receptors in the stroma. Meanwhile, the stromal WNTs, ncWNTs and SEMA3 also target the endothelium. It is also worth noting that lymphatic‐like endothelial cells in the decidua are marked by LYVE1 and prospero homeobox 1 (PROX1), a critical lymphatic‐fating determinant (Figure S4B). This intra‐endothelial transition towards lymphatic fate is reported to be essential for spiral artery remodelling in both mice and rats mediated by the communication between NK cells and the endothelium.^48^ Our results also present compelling evidence that this intra‐endothelial transition is also conserved in humans. The presence of pericytes is noticed for a long time with its function to regulate vasoconstriction. Here, we provide new evidence that pericyte‐derived PDGF would also interact with stromal cells through its receptors. PDGFRα is reported to be broadly expressed in many mesenchymal cells and we prove that PDGFRα is highly expressed in human decidual stromal cells. The function of PDGF in adult tissue is not a major mitogenic factor but more prone to differentiation/migration.^49^ Uterine stromal cells are derived from reproductive mesenchymal cells at the neonatal stage and differentiate to uterine stroma cells characterized with PR expression.^50^, ^51^ It is possible that the PDGF‐PDGFR in pericytes (endothelium) – stroma is very important for the maintenance of haemostasis of stromal cells.

## 5 CONCLUSION

In summary, we present the decidual cell composition of the human maternal‐foetal interface at single‐cell resolution in both normal and RSA decidua, describing a detailed characterization of varied decidual cells, their developmental trajectories, and the communication between stromal cells and other cell populations. This study identifies novel molecular and cellular mechanisms involved with RSA development. The resulting findings will pave the way to improve the diagnosis and prevention of RSA.

## CONFLICT OF INTEREST

There are no competing financial or non‐financial interests regarding this work.

## AUTHOR CONTRIBUTIONS

J.C., H.W and D.C. supervised the overall project design and execution. L.D. and W.D. participated in bioinformatic analyses, wrote the paper and prepared figures. S.Z. and Y.L. performed WB and IHC staining. Y.W. and H.X. performed ISH and IF staining. S.Z., J.T. and L.Z. performed the real‐time PCR detection. S.Z, P.X., L.H., J.T., S.B., Y.L., L.R, L.L., W.D. and M.L. collected the samples and clinical information. Y.Y.L. participated in preparing figures. All authors read and approved the final manuscript.

## Supporting information

Fig S1

Fig S2

Fig S3

Fig S4

Fig S5

Fig S6

Fig S7

Fig S8

Table S1‐2, S4‐6

Table S3

## Data Availability

The datasets generated and analysed in the study are available in the NCBI Gene Expression Omnibus (GEO) and Sequence Read Archive (SRA) (PRJNA672658) and can be accessed upon request. All custom scripts can be accessed upon request to the corresponding author.
