# Supplementary figures and images for: Single‐cell transcriptome analysis reveals defective decidua stromal niche attributes to recurrent spontaneous abortion
Source: Cell Prolif. 2021 Sep 21;54(11):e13125. doi: 10.1111/cpr.13125 (PMC8560595; doi:10.1111/cpr.13125)

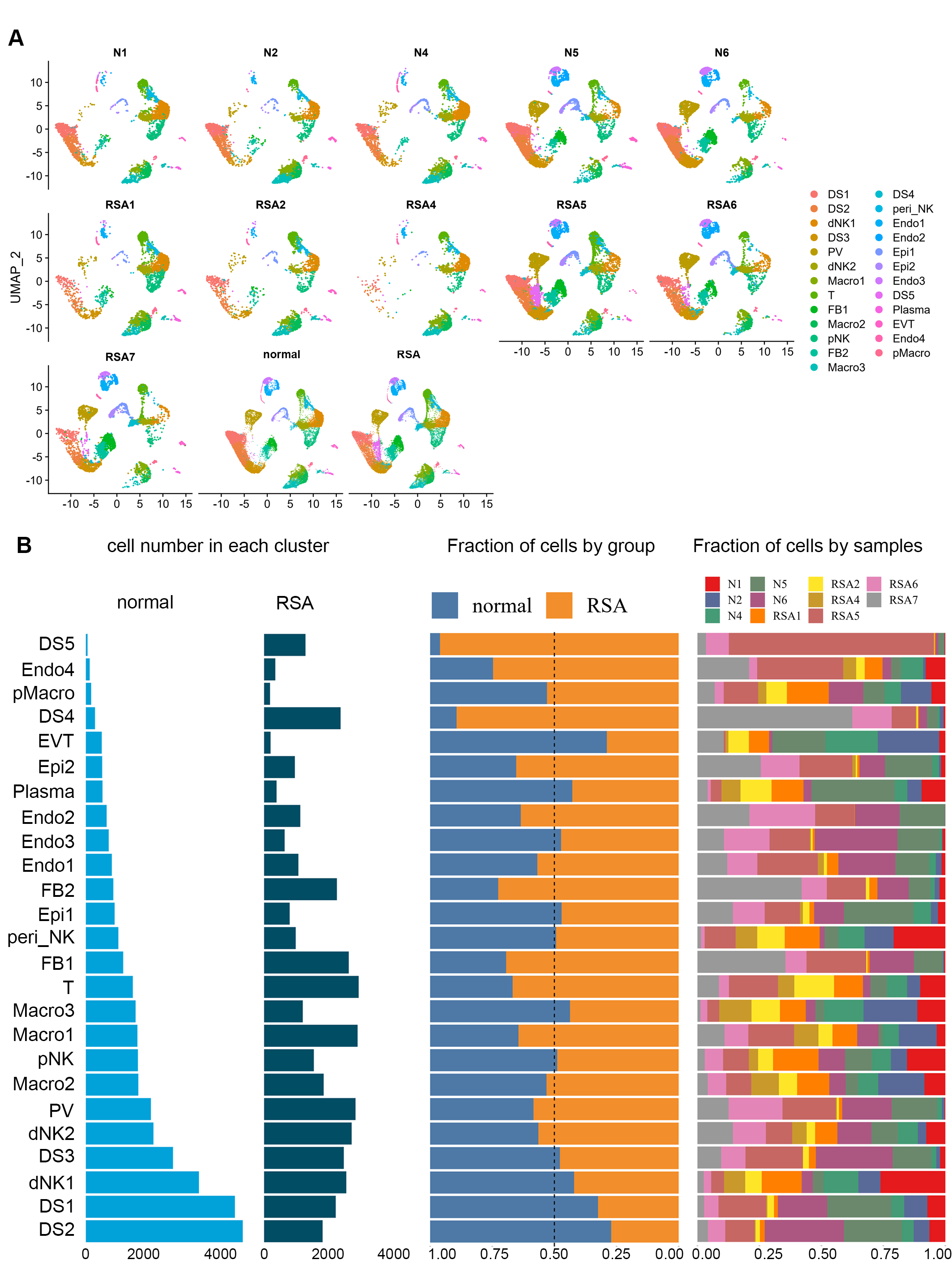

Supplement: Supplementary file 1 — Fig S1 [file CPR-54-e13125-s002.png]

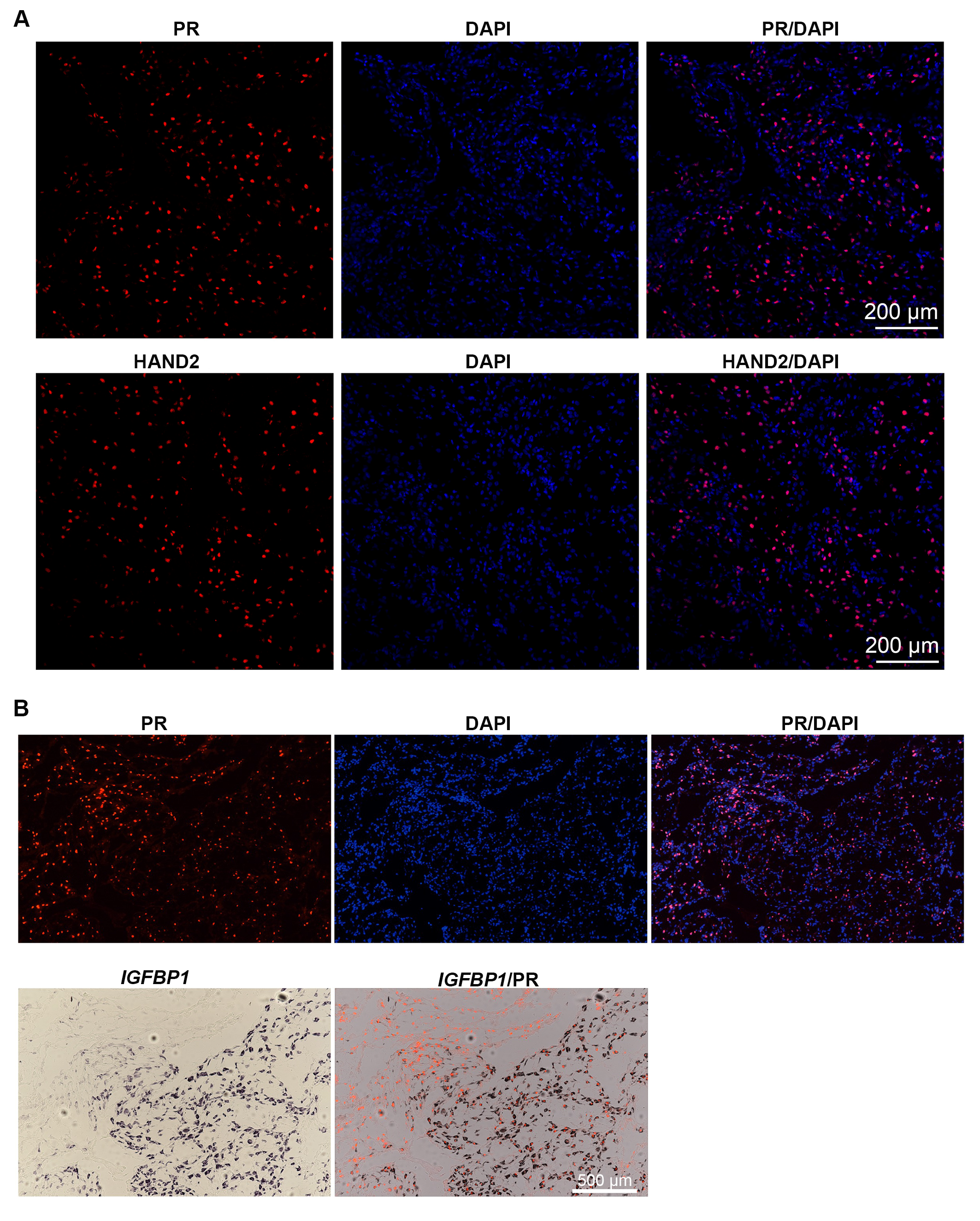

Supplement: Supplementary file 2 — Fig S2 [file CPR-54-e13125-s004.png]

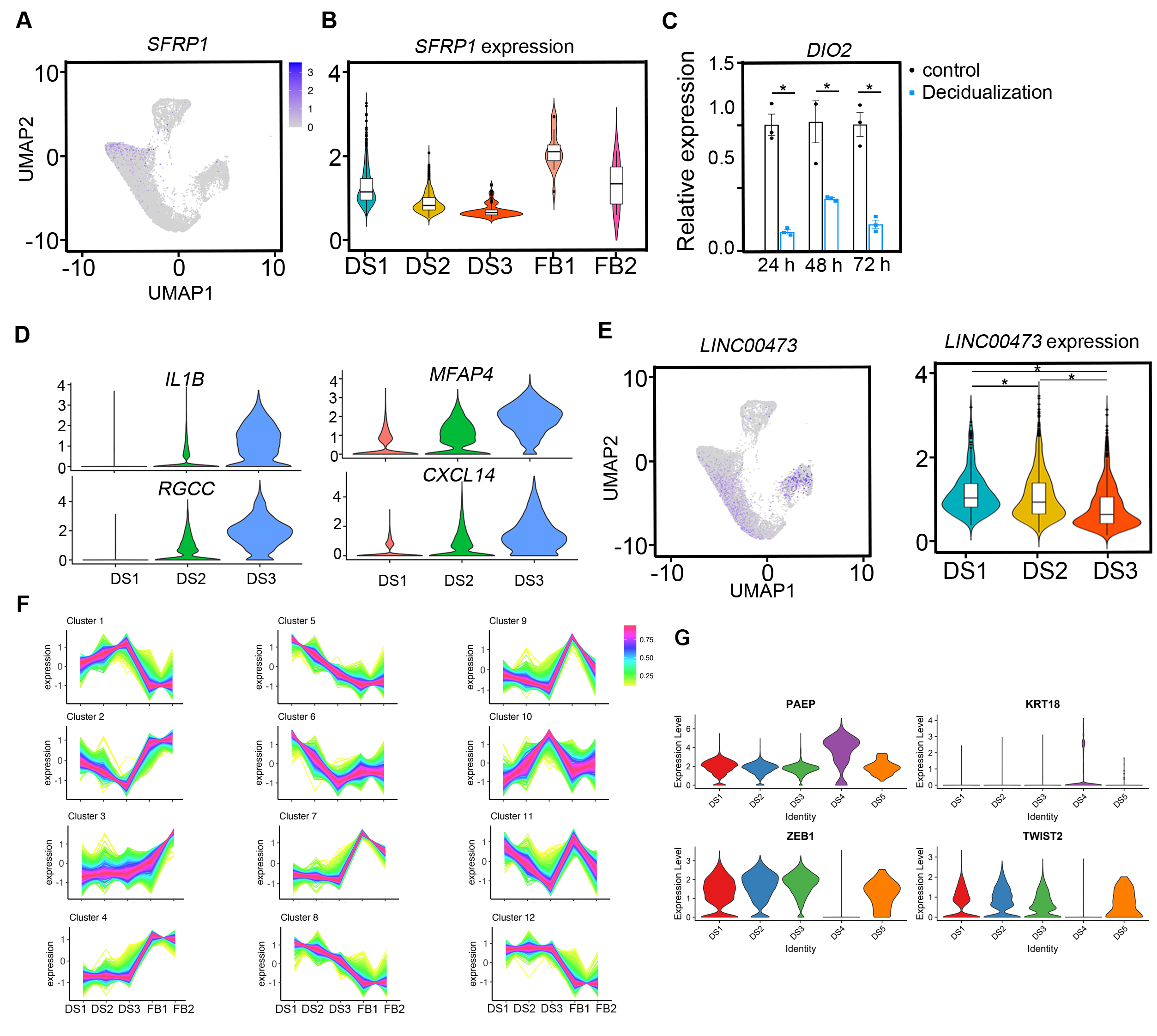

Supplement: Supplementary file 3 — Fig S3 [file CPR-54-e13125-s003.png]

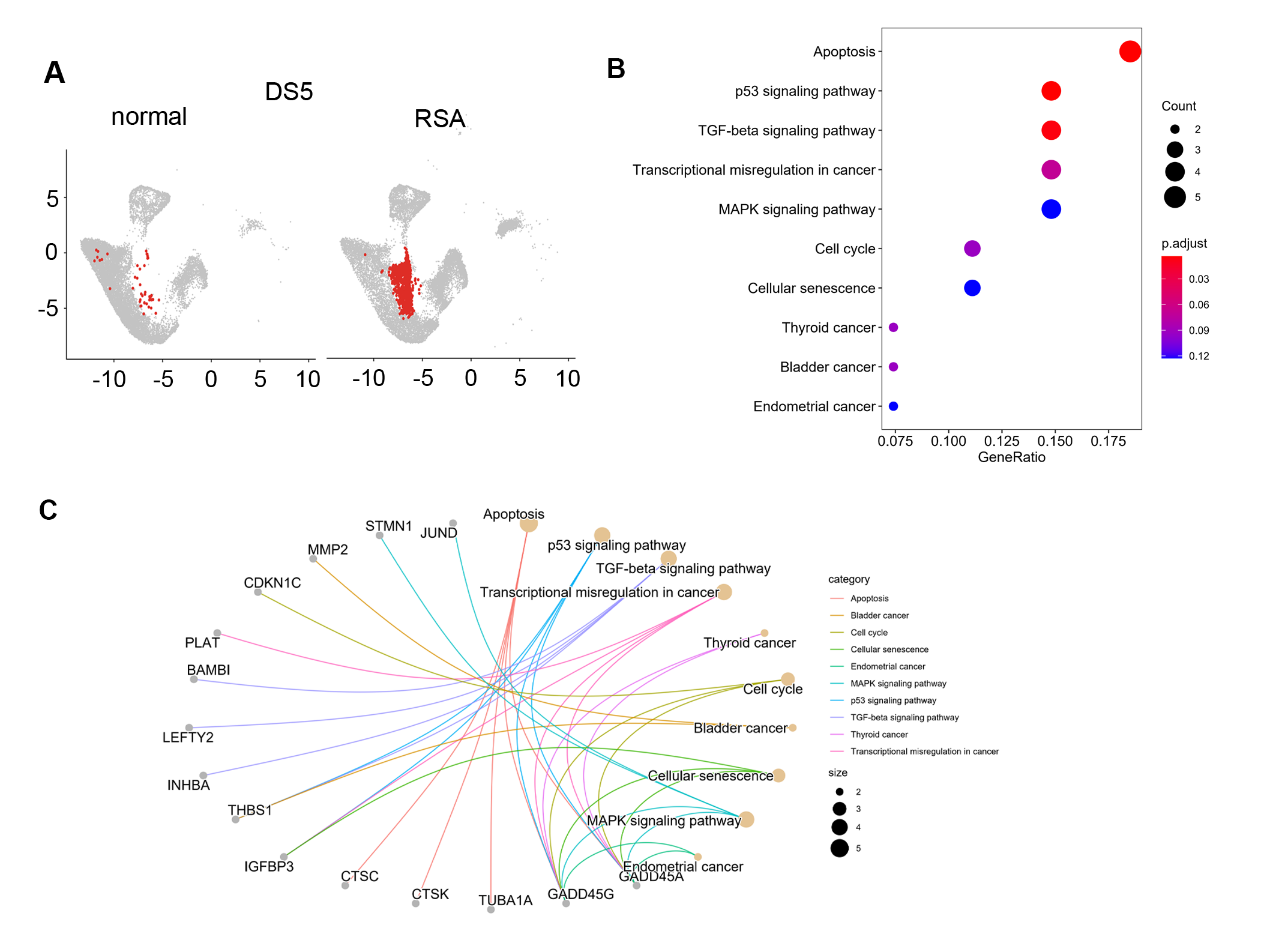

Supplement: Supplementary file 4 — Fig S4 [file CPR-54-e13125-s007.png]

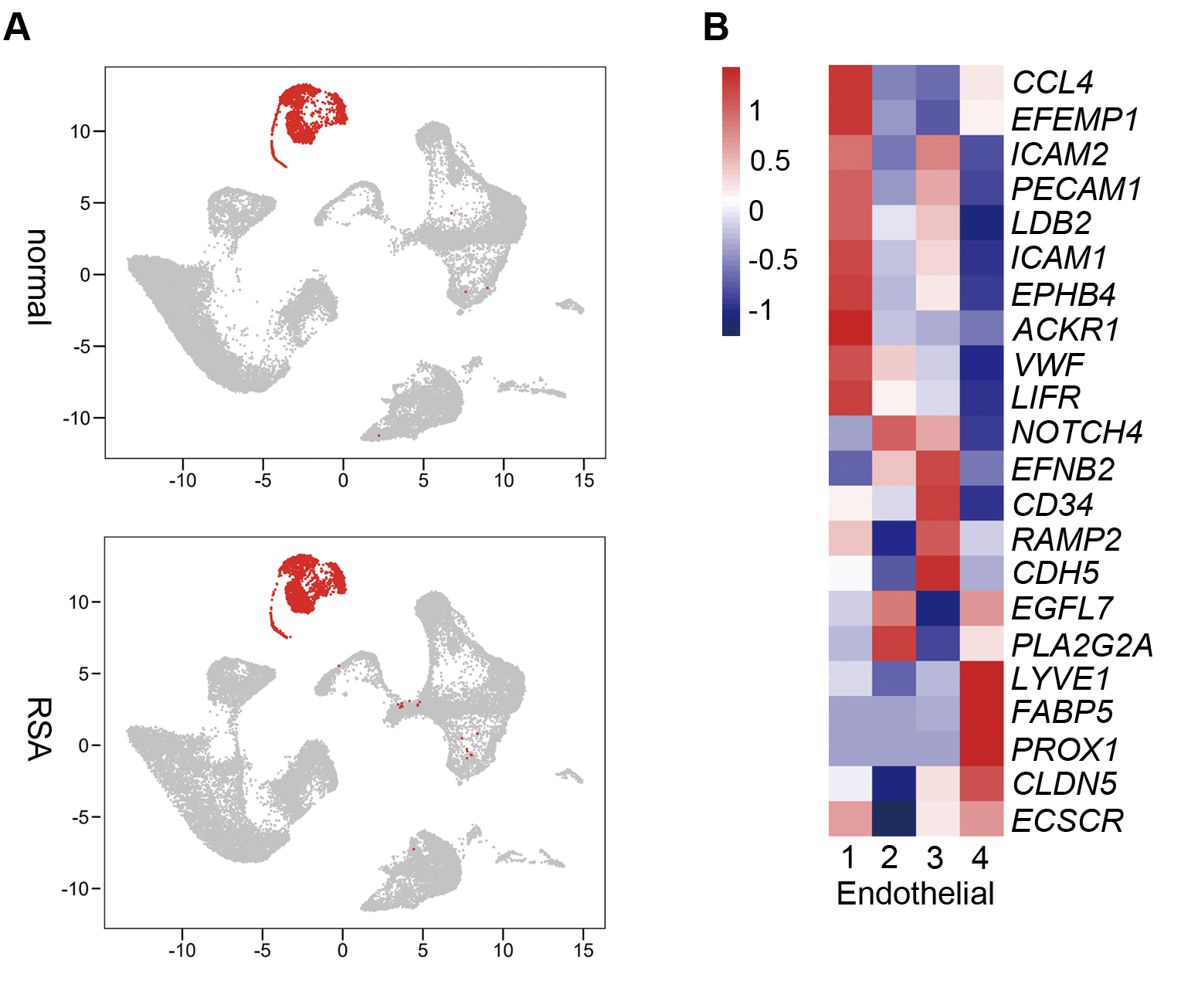

Supplement: Supplementary file 5 — Fig S5 [file CPR-54-e13125-s008.png]

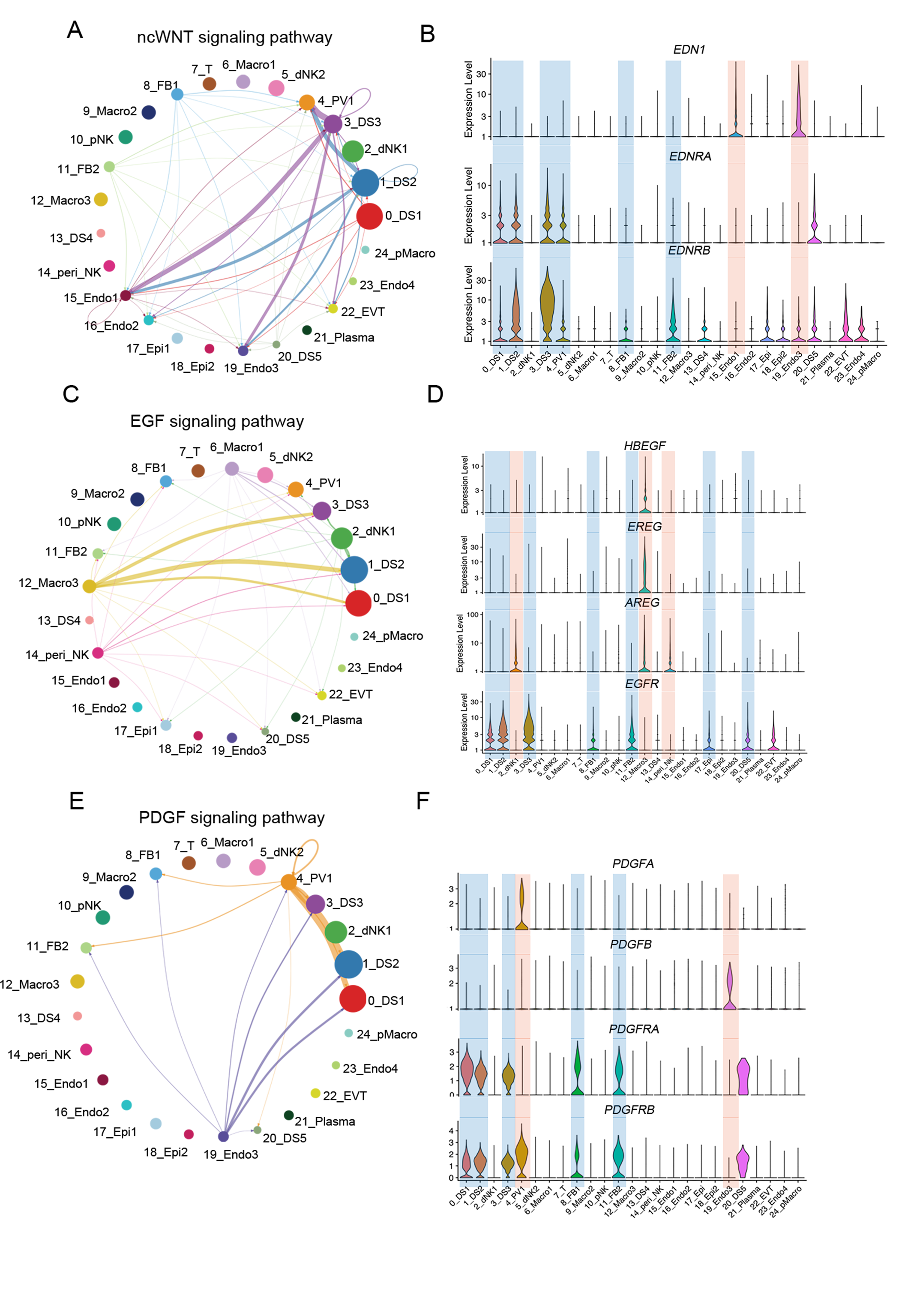

Supplement: Supplementary file 6 — Fig S6 [file CPR-54-e13125-s006.png]

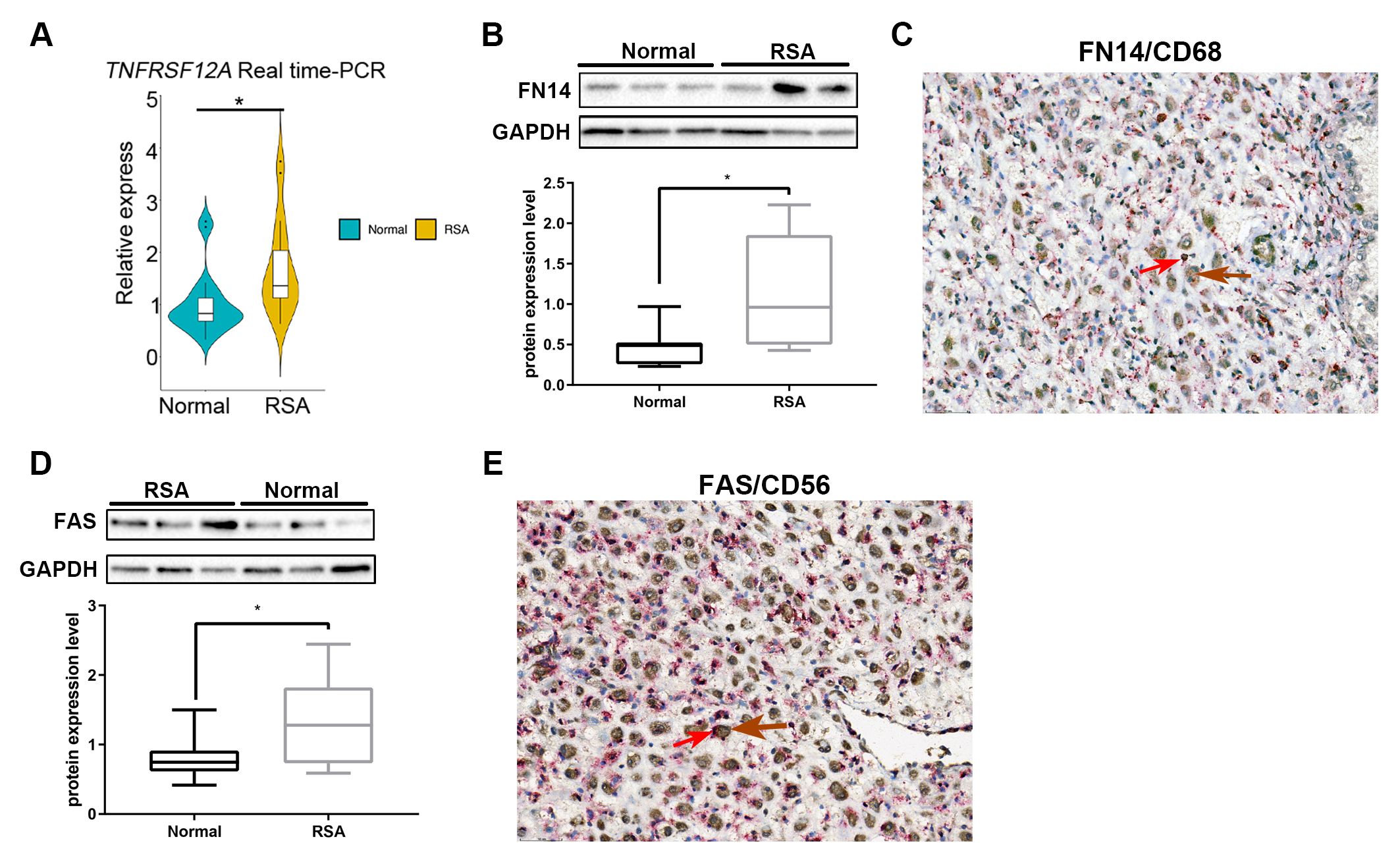

Supplement: Supplementary file 7 — Fig S7 [file CPR-54-e13125-s001.png]

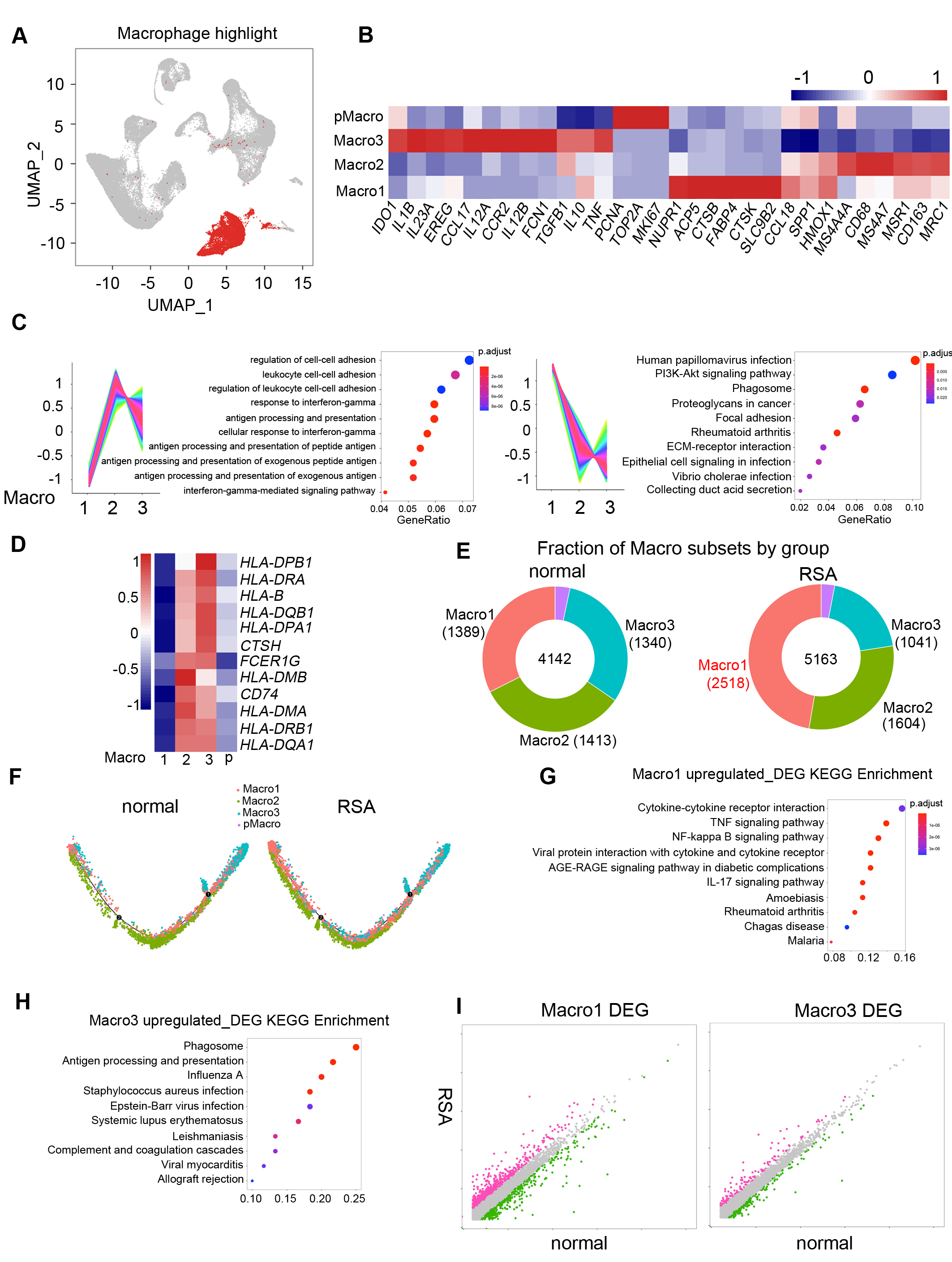

Supplement: Supplementary file 8 — Fig S8 [file CPR-54-e13125-s009.png]
