# Supplementary material for: Single‐cell transcriptome analysis reveals defective decidua stromal niche attributes to recurrent spontaneous abortion
Source: Cell Prolif. 2021 Sep 21;54(11):e13125. doi: 10.1111/cpr.13125 (PMC8560595; doi:10.1111/cpr.13125)
Supplement: Supplementary file 9 — Table S1‐2, S4‐6 [file CPR-54-e13125-s005.docx]

**Supplemental Table S1. Clinical characteristics of women included in scRNA-seq analysis**

|  | **normal (n=5)** | **RSA (n=6)** | ***P*** |
| --- | --- | --- | --- |
| Age (years) | 29.2±7.85 | 33.83±6.24 | 0.303 |
| Gestation age (weeks) | 6.20±1.09 | 6.83±0.75 | 0.285 |
| BMI | 20.25±2.41 | 22.16±2.88 | 0.173 |
| smoking status | None | None |  |
| Previous unexplained pregnancy loss | 0 | 2-5 | <0.001 |
| Parity | 0-2 | 0-1 | 0.824 |
| Chromosomal analysis | - | normal | - |

**Supplemental Table S2. The cell number and quality analysis of scRNA-seq data in each sample**


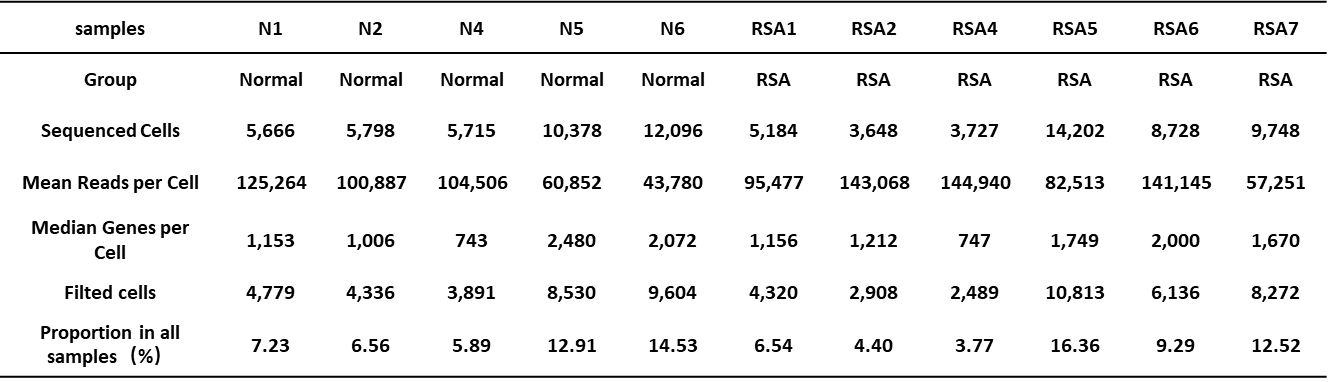


**Supplemental Table S4. Primer sequences for real-time PCR**

| **Primer Name** | **Sequence (5’-3’)** |
| --- | --- |
| *SDHA* | 5’ GAGATGTGGTGTCTCGGTCCAT 3’  5’ GCTGTCTCTGAAATGCCAGGCA 3’ |
| *BAD* | 5’ CCAACCTCTGGGCAGCACAGC 3’  5’ TTTGCCGCATCTGCGTTGCTGT 3’ |
| *GADD45G* | 5’ TTTCGAACCCCAACGAGGAC 3’  5’ TCACTCGGGGAGGGTGATG 3’ |
| *TNFRSF12A*  *DIO2* | 5’ CTCTGAGCCTGACCTTCGTG 3’  5’ GTCTCCTCTATGGGGGTGGT 3’  5’ CCACCCACAAAGAAACAA 3’  5’ TTCCACGTAAATCTCAACAC 3’ |

**Supplementary Table S5** Antibodies used in IHC and IF

| Antibody | Host species | Final concentration | Manufacturer | Catalogue number |
| --- | --- | --- | --- | --- |

| Fn14 | Rabbit | 1:100 | Abcam | ab109365 |
| --- | --- | --- | --- | --- |
| FAS | Rabbit | 1:100 | Sigma | HPA027444 |
| CD68 | Mouse | Ready-to-Use | Agilent(Dako) | GA613 |
| CD56 | Rabbit | 1:100 | LBP | IR040 |
| PR | Rabbit | 1:200 | CST | 8757 |
| HAND2 | Rabbit | 1:200 | Abcam | ab200040 |

**Supplementary Table S6** Antibodies used in WB

| Antibody | Host species | Final concentration | Manufacturer | Catalogue number |
| --- | --- | --- | --- | --- |

| Fn14 | Rabbit | 1:1000 | Abcam | ab109365 |
| --- | --- | --- | --- | --- |
| FAS | Rabbit | 1:1000 | Sigma | HPA027444 |
| GAPDH | Rabbit | 1:1000 | Sigma | SAB4300645 |
